# The Association Between HIV Disclosure Status and Perceived Barriers to Care Faced by Women Living with HIV in Latin America, China, Central/Eastern Europe, and Western Europe/Canada

**DOI:** 10.1089/apc.2016.0049

**Published:** 2016-09-01

**Authors:** Mona Loutfy, Margaret Johnson, Sharon Walmsley, Anna Samarina, Patricia Vasquez, He Hao-Lan, Tshepiso Madihlaba, Marisol Martinez-Tristani, Jean van Wyk

**Affiliations:** ^1^ Women's College Research Institute, Women's College Hospital, University of Toronto, Toronto, Canada.; ^2^ Royal Free Hospital NHS Trust, London, United Kingdom.; ^3^ Immunodeficiency Clinic, Toronto General Hospital, Department of Medicine, University of Toronto, Toronto, Canada.; ^4^ Saint Petersburg City HIV Centre, St. Petersburg, Russia, Saint Petersburg Medical University named after ac. Pavlov I.P., St. Petersburg, Russia.; ^5^ Hospital San Juan de Dios, Santiago de Chile, Chile.; ^6^ Infectious Disease, Guangzhou Eighth People's Hospital, Guangzhou, China.; ^7^ AbbVie, Inc., North Chicago, Illinois.

## Abstract

Generally, women are less likely than men to disclose their HIV status. This analysis examined the relationship between HIV disclosure and (1) perceived barriers to care and (2) quality of life (QoL) for women with HIV. The ELLA (EpidemioLogical study to investigate the popuLation and disease characteristics, barriers to care, and quAlity of life for women living with HIV) study enrolled HIV-positive women aged ≥18 years. Women completed the 12-item Barriers to Care Scale (BACS) questionnaire. QoL was assessed using the Health Status Assessment. BACS and QoL were stratified by dichotomized HIV disclosure status (to anyone outside the healthcare system). Multilevel logistic regression analysis was used to identify factors associated with disclosure. Of 1945 patients enrolled from Latin America, China, Central/Eastern Europe, and Western Europe/Canada between July 2012 and September 2013, 1929 were included in the analysis (disclosed, *n* = 1724; nondisclosed, *n* = 205). Overall, 55% of patients lived with a husband/partner, 53% were employed, and 88% were receiving antiretroviral therapy. Patients who were with a serodiscordant partner were more likely to disclose (*p* = 0.0003). China had a disproportionately higher percentage of participants who did not disclose at all (nearly 30% vs. <15% for other regions). Mean BACS severity scores for medical/psychological service barriers and most personal resource barriers were significantly lower for the disclosed group compared with the nondisclosed group (*p* ≤ 0.02 for all). Compared with the disclosed group, the nondisclosed group reported statistically significantly higher (*p* ≤ 0.03) BACS item severity scores for 8 of the 12 potential barriers to care. The disclosed group reported better QoL. Overall, HIV nondisclosure was associated with more severe barriers to accessing healthcare by women with HIV.

## Introduction

An estimated 16 million women worldwide are living with HIV, which is just over half of the total population of HIV-positive individuals aged 15 years and older.^1^ Women with HIV face additional health issues, potentially poorer overall health outcomes, and unique social and economic challenges compared with their male counterparts.^2–4^

Clinical trials are insufficiently powered to address gender differences in response to therapy, but overall virologic and immunologic responses appear to be similar by gender. Several studies suggest that other clinical outcomes, such as initiating antiretroviral therapy (ART) and maintaining HIV viral load suppression, may be reached less frequently by women than men.^4^ Potential reasons for disparate outcomes include lower levels of engagement with available healthcare resources, reduced likelihood of receiving ART compared with men,^5–7^ delay in ART initiation, complications related to treatment (e.g., higher adverse event rates), and higher rates of treatment discontinuation.^2–4^ Although depression and neurocognitive impairment occur in both women and men living with HIV, women appear to have comparatively higher rates and symptom severity in both conditions.^3,8^

Barriers to accessing care add another dimension to the unique challenges that women living with HIV must confront and have been identified previously.^9–15^ One important barrier for accessing or seeking care is that women are generally more reluctant than men to disclose their HIV status.^16^ Lack of disclosure has been shown to stem from fear of stigma, discrimination, abandonment, or violence.^12^ For example, women may fear involuntary disclosure if they seek HIV treatment and support services within their communities^11^; they may even hesitate to access non-HIV healthcare services, such as those received during labor and delivery.^17^ Furthermore, the elevated incidence of mental health-related issues identified in women versus men with HIV may, in part, underlie the lower levels of HIV/AIDS care seeking seen among women with HIV^4,8,18^ and may also play a role in the relationship between disclosure and barriers to accessing care.

Disclosure of HIV status is acknowledged to be an important possibly difficult and complex process^19^; however, the available evidence finds that HIV disclosure may be associated with improved health outcomes and positive behaviors, such as improved adherence to antiretroviral medication.^19–22^ Given its central role in the health and well-being of women with HIV, it is important to better understand the associations between disclosure and barriers to accessing care and the factors that may engender or hinder disclosure. In this subanalysis of a large multicountry, cross-sectional noninterventional study [EpidemioLogical study to investigate the popuLation and disease characteristics, barriers to care, and quAlity of life for women living with HIV (the ELLA study)], we examined the associations between disclosure of HIV status and perceived barriers to care and quality of life (QoL).

## Methods

### Study design

The ELLA study was conducted across four global geographic regions [Latin America (LA), China, Central/Eastern Europe (CEE), and Western Europe/Canada (WEC)] and included 114 sites in 27 countries. Study enrollment occurred between July 2012 and September 2013. The primary objective, which was to describe the prevalence and correlates of barriers to healthcare faced by women living with HIV, has been described previously.^10^ The focus of the current subanalysis is to describe the status of HIV disclosure for the ELLA study participants and the relationships between disclosure and perceived barriers to healthcare.

### Participants and procedures

Participants were HIV-positive women aged ≥18 years who had been diagnosed at least 3 months before enrollment. Women were given the opportunity to participate in the study (nonrandom sequential sampling frame design) while attending a routine follow-up clinic visit. All participants signed an informed consent to participate in the study and to use and disclose personal health information. To ensure a robust data set for analysis, women who answered six or more Barriers to Care Scale (BACS) items were included in the calculation of overall BACS scores. For women with six or fewer BACS items missing, the mean substitution method was used to impute scores for the missing items.

Women completed questionnaires at a single time point. Study investigators provided participants' demographic, social, and educational background information; data related to HIV infection and comorbidities; relevant medical history; and utilization of healthcare services. Healthcare services included consultation and experience with obstetrics/gynecology services, last Pap smear and/or mental health services in the past 12 months, and attendance at scheduled clinic visits in the past 12 months.

### Exposure of interest

Disclosure was the exposure of interest and was descriptively summarized by its extent as an ordinal variable as follows: (1) not disclosed to anyone outside the healthcare system; (2) disclosed to close/intimate relations; (3) disclosed to extended relations; and (4) complete disclosure. To identify factors associated with disclosure of HIV status, disclosure (any extent) was dichotomized versus no disclosure to anyone outside the healthcare system.

### Endpoints of interest

The 12-item BACS was one of the endpoints of interest.^23^ Rated barriers were (1) long distances to medical facilities; (2) medical personnel who decline to provide direct care to a person with HIV; (3) lack of healthcare professionals (HCPs; trained and competent in HIV care); (4) lack of transportation to access needed services; (5) shortage of mental healthcare personnel; (6) lack of psychological support groups; (7) level of knowledge about HIV among citizens in the community; (8) stigma of community residents against persons living with HIV; (9) lack of employment opportunities; (10) lack of supportive and understanding work environments; (11) personal financial resources; and (12) lack of adequate and affordable housing. Each item was rated on a 4-point Likert scale (1 = no problem at all, 2 = very slight problem, 3 = somewhat of a problem, 4 = major problem) to indicate the extent to which each barrier made it difficult to receive the care, services, or opportunities patients wished to obtain. Scores ≥2 were considered to be significant.

A second endpoint of interest was QoL, assessed using a Health Status Assessment, which included two general QoL measures, a general health item (5-point scale: excellent, very good, good, fair, and poor) and a current health status assessment (scale from 1 to 100, with 0 = death or worst possible health and 100 = perfect or best possible health).^24^

### Statistical analysis

Sociodemographic and exposure data and endpoints of interest were summarized using frequencies and proportions for categorical variables and medians with interquartile ranges for continuous variables. Chi-square and Wilcoxon–Mann–Whitney tests were used for between-group comparisons of categorical and continuous variables, respectively.

Multilevel logistic regression analysis was conducted to identify factors that were associated with disclosure prevalence, dichotomized as outlined above. Factors assessed in the multilevel logistic regression included region, age group, immigration status, area of residence, living status, partner HIV status, presence of friend/family support, years of formal education, employment status, payment plan, number of children, number of children living at home, last viral load, last CD4^+^ T-cell count, risk factors for acquiring HIV, time from diagnosis to enrollment, ART use, number of comorbidities, smoking history, AIDS diagnosis, change in treatment facility over previous 12 months, number of appointments per year, and number of missed appointments. Site-specific factors assessed included adherence to therapy guidelines, type of site, availability of additional clinic services, availability of child care and transportation services, availability of female therapies, average visit frequency, routine human papillomavirus testing, mental health assessment, BACS scores, and health assessment scores.

A univariate multilevel logistic regression was conducted first, followed by a multivariate multilevel regression. The multivariate analysis, which included the factors from the univariate analysis that were associated with disclosure prevalence at *p* < 0.20, determined those that were associated at *p* < 0.05, while controlling for other significant covariates. The stepwise selection method was used to develop the model. Effects are entered into and removed from the model in such a way that each forward selection step may be followed by one or more backward elimination steps. The stepwise selection process terminates if no further effect can be added to the model or if the effect just entered into the model is the only effect removed in the subsequent backward elimination. Last, an interaction term between the variable for geographic area and each significant factor identified through multivariate analysis was tested to assess whether the effect of the factor on disclosure prevalence was different between geographic areas. In the univariate and multivariate analyses, the measure of association of each factor with disclosure prevalence was the odds ratio (OR) of any extent versus no disclosure, presented with 95% confidence interval (CI).

Analyses of interest included the relationship between HIV disclosure status and prevalence and severity scores for individual BACS items and between HIV disclosure status and QoL. Between-group comparisons were assessed using the Wilcoxon–Mann–Whitney test (for continuous variables) or the chi-square test (for categorical variables).

## Results

### Disclosure and study population

**FIG. 1.**
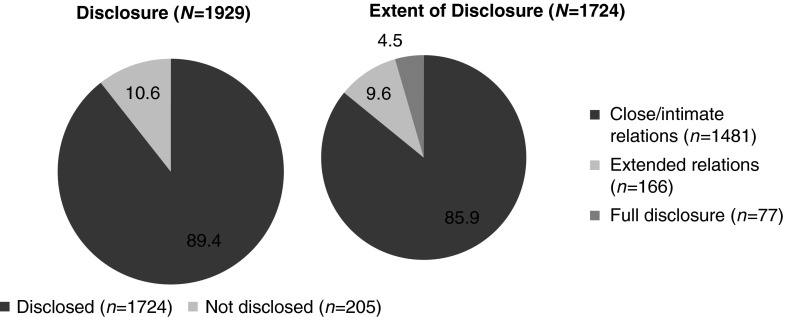
HIV disclosure status.

Among the 1945 participants who enrolled in ELLA, 1929 were included in the disclosure analysis (14 were excluded because of protocol violations and 2 were missing disclosure data). Most women (89.4%; *n* = 1724) had disclosed their HIV status to some extent outside the healthcare system; 85.9% had disclosed their HIV status to only close/intimate relations, 9.6% disclosed to extended relations, and 4.5% had fully disclosed (Fig. 1). Across regions, the most prevalent type of disclosure was to close/intimate relations. Notably, China, where this type of disclosure was lowest (68%), had a disproportionately higher percentage of participants who did not disclose at all (nearly 30% vs. <15% for other regions; Table 1).

**Table 1.** Disclosure by Region

*Disclosure type,*^a^ n *(%)*	*China (*n* = 120)*	*Central and Eastern Europe (*n* = 532)*	*Latin America (*n* = 519)*	*Western Europe and Canada*^b^*(*n* = 760)*	*Total (*N* = 1931)*
Not disclosed	35 (29)	39 (7)	35 (7)	96 (13)	205 (11)
*p* Value^c^	<0.0001	0.0060	0.0048	—	—
Disclosed to close/intimate relations	81 (68)	438 (82)	415 (80)	547 (72)	1481 (77)
*p* Value^c^	0.6536	<0.0001	0.0048	—	—
Disclosed to extended relations	3 (3)	46 (9)	54 (10)	63 (8)	166 (9)
*p* Value^c^	0.0701	0.9999	0.4116	—	—
Full disclosure	1 (1)	9 (2)	15 (3)	52 (7)	77 (4)
*p* Value^c^	0.0422	<0.0001	0.0051	—	—
Missing	0	0	0	2 (<1)	2 (<1)

^a^ Percentages may not total 100% because of rounding.

^b^ Comparator.

^c^ Chi-square (categorical variables).

Sociodemographic characteristics are summarized in Table 2. Several characteristics were associated with disclosure to anyone outside the healthcare system; participants who disclosed were younger, more likely to live with a partner, and more likely to receive support from family or friends. Furthermore, participants who disclosed were more likely to have been born in their country of residence and were also more likely to be with a serodiscordant partner. Notably, 6% (67/1066) of participants who reported living with a partner or husband had not disclosed and 40% (27/67) of women who were living with a partner or husband and serodiscordant had not disclosed their HIV status to anyone outside the healthcare system, including that partner or husband (Table 2).

**Table 2.** Demographic and Clinical Characteristics by HIV Disclosure Status

*Characteristic,* n *(%)*	*No disclosure (*n* = 205)*	*Any disclosure*^a^*(*n* = 1724)*	*Total (*N* = 1929)*	p^b^
Age,^c^ years	41 (15)	39 (17)	39 (16)	0.0080
Residence				0.7050
Rural	37 (18)	293 (17)	330 (17)	
Urban	168 (82)	1431 (83)	1599 (83)	
Living status				<0.0001
Alone	86 (42)	328 (19)	414 (22)	
With others	52 (25)	397 (23)	449 (23)	
With partner/husband	67 (33)	999 (58)	1066 (55)	
Serodiscordant with partner^d^	27 (40)	486 (49)	513 (48)	0.0003
Employed	119 (58)	894 (52)	1013 (53)	0.2164
Born in country of residence	139 (68)	1382 (80)	1521 (79)	0.0008
Family/friend support	48 (23)	1111 (64)	1159 (60)	<0.0001
Time from diagnosis to enrollment, years	6.3 (7.9)^c^	8.0 (9.6)^c^	7.8 (9.4)^c^	0.0215
<1	15 (7)	103 (6)	118 (6)	
1–5	78 (38)	538 (31)	616 (32)	
>5–10	57 (28)	427 (25)	484 (25)	
>10	46 (22)	587 (34)	633 (33)	
Unknown	9 (4)	69 (4)	78 (4)	
Latest viral load <50 copies/mL	126 (62)	985 (57)	1111 (58)	0.4036
Last recorded CD4^+^ count,^c^ cells/mL	482 (331)	517 (395)	513 (386)	0.9406
Use of antiretroviral therapy				0.1526
Never	20 (10)	136 (8)	156 (8)	
Previous	3 (2)	67 (4)	70 (4)	
Current	182 (89)	1521 (88)	1703 (88)	
Comorbidities >10% in total population				
Anxiety/depression	36 (18)	316 (18)	352 (18)	
Hepatitis C	16 (8)	268 (16)	284 (15)	

^a^ Disclosure to close/intimate relations (*n* = 1481), to extended relations (*n* = 166), or full disclosure (*n* = 77).

^b^ Chi-square (categorical variables) or Wilcoxon–Mann–Whitney (continuous variables) test, disclosed versus nondisclosed.

^c^ Median (interquartile range).

^d^ For women living with a partner (no disclosure, *n* = 27; any disclosure, *n* = 486; total, *n* = 513).

Disease characteristics were not associated with disclosure, with the exception of the overall distribution of time from diagnosis to enrollment in the study (*p* = 0.02; Table 2). Most participants, regardless of disclosure status, enrolled in ELLA >1 year after their diagnosis, and the vast majority (almost 90%) reported current use of ART.

### Logistic regression analysis of factors associated with disclosure

**FIG. 2.**
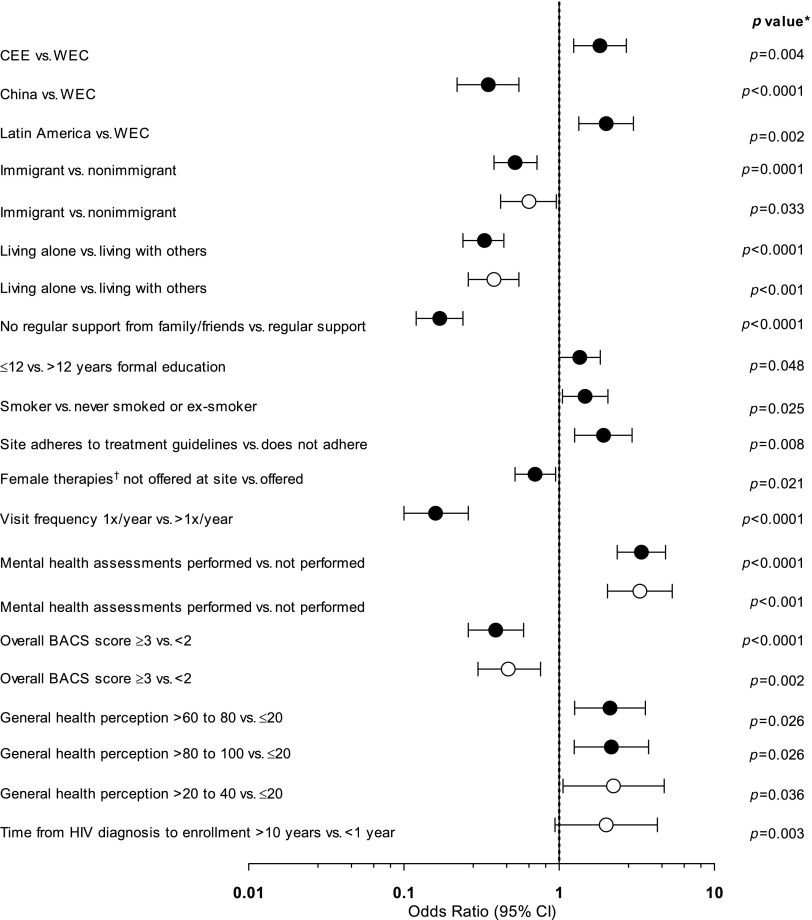
Univariate and multivariate logistic regression analysis of factors associated with HIV disclosure status. Univariate, solid circles; multivariate, open circles. *Adjusted for multiple testing using the Benjamini and Yekutieli (2001) procedure.^25^^†^Contraceptive, mental health-related, or hormonal therapies. BACS, Barriers to Care Scale; CEE, Central/Eastern Europe; WEC, Western Europe/Canada.

In univariate analyses, factors associated with higher odds of disclosure included living in CEE versus WEC, living in LA versus WEC, having ≤12 versus >12 years of formal education, being a current smoker, adherence of the clinic site to treatment guidelines, performance of mental health assessments, and more favorable assessments of general health (Fig. 2; solid circles). Factors statistically significantly associated with lower odds of disclosure were living in China versus WEC, being an immigrant, living alone, not having regular support from family/friends, not having female therapies offered at the clinic site, a visit frequency of once per year versus greater than once per year, and an overall BACS score ≥3 versus <2.

In the multivariate analysis, factors associated with higher odds of disclosure included having a general health perception score >20–40 (on a scale of 1–100) versus ≤20 (OR, 2.2 [95% CI, 1.1–4.8]; *p* = 0.036), having a longer time from HIV diagnosis to enrollment (>10 years versus <1 year; OR, 2.0 [95% CI, 0.9–4.3]; *p* = 0.003), and having had a mental health assessment performed (OR, 3.3 [95% CI, 2.1–5.4]; *p* < 0.001) (Fig. 2; open circles). Factors associated with lower odds of disclosure were being an immigrant (OR, 0.6 [95% CI, 0.4–1.0]; *p* = 0.033), an overall BACS score ≥3 versus <2 (OR, 0.5 [95% CI, 0.3–0.8]; *p* = 0.002), and living alone versus living with others (OR, 0.4 [95% CI, 0.3–0.6]; *p* < 0.001).

Multivariate analysis revealed that by geographic area, CEE was significantly different from WEC regarding HIV disclosure (*p* = 0.01). Regular family/friends' support had a significant effect on HIV disclosure (*p* < 0.001), and the interaction term indicates that regular family/friends' support affected disclosure differently in CEE and WEC (*p* = 0.002), as well as LA and WEC (*p* = 0.005).

### Barriers to care

**FIG. 3.**
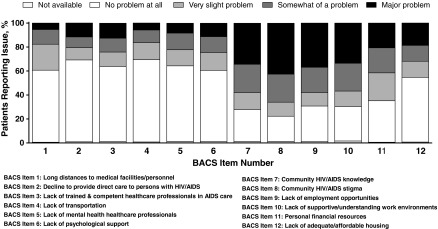
Prevalence of barriers to care (total population).

Results of the BACS for the overall population (*N* = 1929) found distinct patterns in prevalence among the scale items (Fig. 3). Most participants reported no problem at all with long distances to medical facilities/personnel (60.1%), medical personnel declining medical care (68.3%), lack of trained and competent HCPs in AIDS care (62.7%), lack of transportation to access services (68.6%), lack of mental health HCPs (63.0%), lack of psychological support groups (59.5%), or lack of adequate and affordable housing (54.0%). However, a majority of participants overall (77.7%) noted concern about community HIV/AIDS stigma, with 42.6% noting it to be a major problem. Community knowledge about HIV/AIDS was a problem for 72.2% of participants overall and was rated as a major problem by 34.3%. Other barriers noted as problematic by the majority of participants were a lack of supportive/understanding work environments (a major problem for 33.5%), lack of employment opportunities (a major problem for 36.9%), and personal financial resources (a major problem for 20.6%).

### Disclosure and barriers to healthcare

**FIG. 4.**
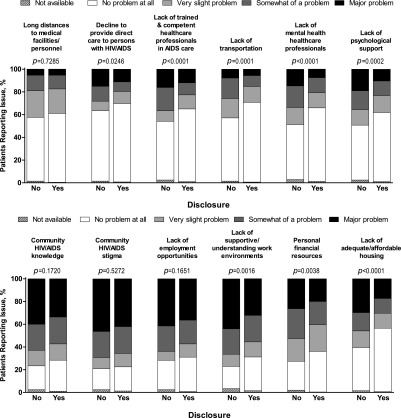
Prevalence of barriers to care by HIV disclosure status.

**FIG. 5.**
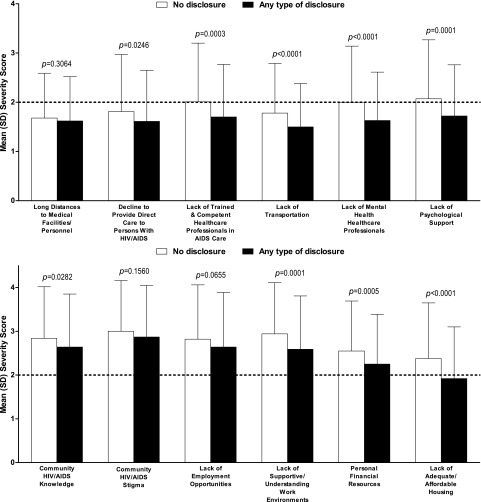
Severity of barriers to care by HIV disclosure status.

Eight items on the BACS were more frequently reported as problematic (Fig. 4) and nine items were rated as more severe (Fig. 5) by participants who did not disclose their HIV status. For participants who did not disclose, the prevalence and the severity of the following barriers were significantly higher: medical personnel who decline to provide direct care to a person with HIV/AIDS, lack of HCPs adequately trained and competent in AIDS care, lack of transportation, shortage of mental health HCPs, lack of psychological support, lack of supportive and understanding work environments, personal financial resources, and lack of adequate or affordable housing. The severity score, but not prevalence, of community HIV/AIDS knowledge as a barrier was significantly higher in participants who disclosed to any extent.

Community HIV/AIDS stigma and the lack of community HIV/AIDS knowledge, employment opportunities, supportive work environments, and personal financial resources were reported as severe (i.e., mean severity score >2) barriers to care by both disclosure groups.

### Quality of life

**FIG. 6.**
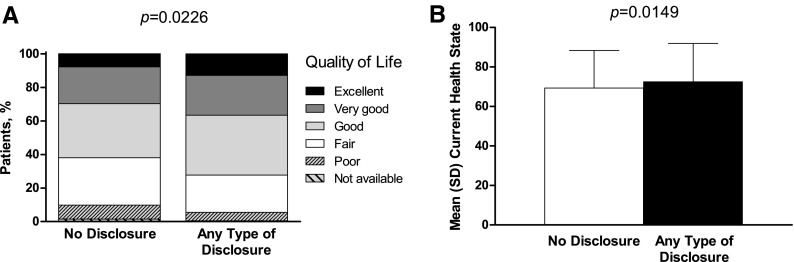
Participant-rated quality of life by HIV disclosure status for **(A)** general health and **(B)** current health status.

Participants who disclosed their HIV status (any extent) versus those who did not were significantly more likely to report better QoL on both the general health questionnaire (*p* = 0.0226; Fig. 6A) and the current health status assessment (disclosed vs. not disclosed, mean ± standard deviation): 72.5 ± 19.5 vs. 69.3 ± 19.0 (*p* = 0.0149; Fig. 6B). Of the women who disclosed, 12.8% (220/1724) reported that their general health was excellent, compared with 7.8% (16/205) of women who did not disclose. Responses to the following QoL survey items differed significantly between participants who disclosed and those who did not: trouble with attention on activities (*p* < 0.0184), felt calm and peaceful (*p* < 0.0246), tired (*p* < 0.0177), enough energy (*p* < 0.0002), happy (*p* < 0.0007), and feeling bad (*p* < 0.0052).

## Discussion

This analysis of the large observational ELLA study provides global perspectives on the disclosure of HIV status among women living with HIV and its correlates and the associations of disclosure with barriers to accessing care and QoL. Although most women across regions disclosed HIV to some extent, a very low proportion (<5%) fully disclosed their diagnosis. When HIV status was disclosed, it was mostly limited to intimate relations (77%). Women living in China had lower levels of disclosure compared with those living in WEC. We previously reported that in this study population, community stigma severity was greater in China than in WEC,^26^ which may explain, at least in part, the lower odds of disclosure. Living in CEE or LA was associated with higher odds of disclosure compared with living in WEC. As previously reported,^10^ the higher rate of immigration rates in WEC (42.6%) compared with CEE (4.7%) and LA (11.8%) may account, at least in part, for the lower rate of disclosure in WEC. Disclosure was significantly associated with both barriers to accessing care and QoL. Our results are consistent with a study conducted in the United States in which participants who did not disclose their HIV status were observed to be twice as likely to have poor retention in care compared with those who reported broad disclosure.^5,27^

Multivariate analysis revealed two factors most strongly associated with disclosure. Living alone was associated with nondisclosure; given that disclosure is overwhelmingly most frequent to intimate relations, this finding is not unanticipated. Anxiety and/or depression were common and reported at similar levels regardless of disclosure; however, having had a mental health assessment performed within the past 12 months was associated with disclosure of HIV. Walstrom et al.^28^ showed that utilization of mental health services, including support group participation, may lead to increased ART adherence and increased HIV status disclosure, benefiting psychological functioning and physical health. These findings and the other significant findings of immigrant status, higher BACS score, higher self-rating of general health, and longer duration of HIV/AIDS should be further investigated with regard to association with disclosure, particularly because several of these factors can potentially be modified if addressed in clinical practice. Although other researchers have noted that disclosure may be associated with improved health outcomes,^19^ we did not find a relationship between disclosure and clinical variables such as suppression of viral load and CD4^+^ T-cell counts. This may be because most women, regardless of disclosure status, were being treated with highly efficacious, well-tolerated ART regimens. The relationship of other clinical outcomes with disclosure, including detection of other comorbidities such as cervical cancer, should be an area of further investigation. Future investigations should also examine the involvement of, and disclosure to, services outside the primary healthcare provider.

These data suggest that there is an important relationship between HIV disclosure (any extent) and the prevalence and severity of barriers to care. Women who did not disclose their HIV status outside the healthcare system reported higher (worse) BACS scores regarding lack of affordable housing, finances, supportive environment, mental healthcare, and transportation. Certain barriers, including community stigma, community HIV/AIDS knowledge, and lack of employment opportunities, were highly prevalent and/or severe regardless of disclosure status. Notably, patient-reported assessments of QoL were lower among participants who did not disclose HIV status, although the study design does not allow us to understand the direction of this relationship. However, it is plausible that disclosure may provide a psychological benefit by reducing anxiety due to fear of accidental disclosure. Disclosure likely also provides greater opportunity for women with HIV to seek assistance from friends and family with healthcare-related matters, thereby improving mental and physical QoL. Our results, demonstrating a correlation between mental health evaluation and disclosure rates and between disclosure and QoL, suggest that to achieve maximum QoL, women with HIV should be provided with referrals for mental health evaluation.

Several study limitations must be noted. Sites involved in ELLA were invited and participation was voluntary. No effort was made to balance sites with different levels of services offered. Only women able to reach healthcare facilities and those who could read and write participated in the study; the extent of disclosure, barriers in accessing care, and QoL of women who could not participate remain unknown. Countries were grouped by geographic region, within which participant characteristics and the HIV epidemic generally may vary. Such heterogeneity may produce bias in analysis. Additionally, China was the only Asian country included, which may limit generalizability of results to other Asian countries. Africa has the highest number of HIV-positive women worldwide; however, it was not represented in this study. The BACS questionnaire has been validated in HIV-positive patients in the United States only; however, dual translation (forward and backward) was provided in all questionnaires to eliminate language barriers and minimize misinterpretation. Finally, men living with HIV were not included in ELLA, therefore precluding comparisons by gender. Results of this analysis of the ELLA study find that most women living with HIV disclose their status most often only to close relations. Furthermore, it appears that women who disclose have a lower prevalence and severity of barriers to accessing care and a higher QoL. Although women living with HIV may choose to limit the extent that they share knowledge of their illness, disclosure itself may be associated with improved well-being. This relationship and the factors identified in this analysis that may facilitate or hinder disclosure should be further investigated.
